# ChemProps: A RESTful API enabled database for composite polymer name standardization

**DOI:** 10.1186/s13321-021-00502-6

**Published:** 2021-03-12

**Authors:** Bingyin Hu, Anqi Lin, L. Catherine Brinson

**Affiliations:** Department of Mechanical Engineering and Materials Science, Duke University, Durham, NC 27708 USA

**Keywords:** Polymers, SMILES, API, Materials Informatics, NanoMine, Database, Optimization

## Abstract

The inconsistency of polymer indexing caused by the lack of uniformity in expression of polymer names is a major challenge for widespread use of polymer related data resources and limits broad application of materials informatics for innovation in broad classes of polymer science and polymeric based materials. The current solution of using a variety of different chemical identifiers has proven insufficient to address the challenge and is not intuitive for researchers. This work proposes a multi-algorithm-based mapping methodology entitled ChemProps that is optimized to solve the polymer indexing issue with easy-to-update design both in depth and in width. RESTful API is enabled for lightweight data exchange and easy integration across data systems. A weight factor is assigned to each algorithm to generate scores for candidate chemical names and optimized to maximize the minimum value of the score difference between the ground truth chemical name and the other candidate chemical names. Ten-fold validation is utilized on the 160 training data points to prevent overfitting issues. The obtained set of weight factors achieves a 100% test accuracy on the 54 test data points. The weight factors will evolve as ChemProps grows. With ChemProps, other polymer databases can remove duplicate entries and enable a more accurate “search by SMILES” function by using ChemProps as a common name-to-SMILES translator through API calls. ChemProps is also an excellent tool for auto-populating polymer properties thanks to its easy-to-update design.

**Supplementary Information:**

The online version contains supplementary material available at 10.1186/s13321-021-00502-6.

## Introduction

Significant advances in computing power in the past decade have given birth to many data-driven approaches including Materials Informatics, which facilitates understanding of processing-structure–property relationships and is a cornerstone in the materials design process [1–4]. However, as Ramprasad et al. points out, Materials Informatics requires data to be reliable, uniform and stored in a controlled manner [5]. This seemingly simple requirement has posed many challenges for polymeric materials data due to prevalent use of different naming conventions and abbreviations for polymers. While the various aliases are fluently acceptable for humans, they confound attempts to curate data in the robust and consistent manner essential for indexing into databases for Materials Informatics, which is considered a major impediment for the adoption of machine learning techniques [6, 7].

The correctness of data indexing can impact many aspects of a data resource. Because of the lack of uniformity in expression of polymer names in publications and data sets, exploration of the data via search and visualization tools becomes problematic, leading to difficulties in using a polymer data resource as a viable tool in data driven discovery. For example, the results returned for searching for Poly(methyl methacrylate) (PMMA) on Reaxys [8], which is a powerful database with properties and reaction data available for a wide range of substances including polymers, varies by the expression of PMMA.^1^

NanoMine is another example. NanoMine is an ontology-enabled open source data resource for the polymer nanocomposite community. The visualization tool of NanoMine is hampered by the polymer nomenclature in terms of the existence of multiple different labels for the same polymer. As a result, users cannot perform simple operations such as filtering the plot by polymer matrix readily. A tool resolving the nomenclature discrepancy is useful to polymer data resources like NanoMine and the whole community.

Researchers have made efforts in polymer indexing through use of chemical identifiers which fall into two categories. The first is source-based such as PubChem CIDs [9], ChEBI IDs [10] and CAS numbers [11]. These number-form identifiers are generated by common online chemical platforms. Regardless of the data quality issue, these chemical identifiers are usually not favorable for cross-database studies due to the source dependency [12]. The second category of chemical identifiers are source independent, such as IUPAC names, SMILES notations [13] and InChI strings [14]. However, Akhondi et al. found out that the consistency of the source independent chemical identifiers between data sources counterintuitively varies significantly [15]. A recent effort entitled BigSMILES [16] was made to build upon the SMILES notation for better representation of the stochastic nature of polymers. BigSMILES could be a solution to the inconsistency use of chemical identifiers once it is canonicalized, which is still ongoing. Even when a polymer database is correctly indexed, it is not intuitive for users to search for chemicals with chemical identifiers. In many cases, researchers cannot read the chemical identifiers since these expressions are not typically used regularly in communications or record keeping. Consider “polystyrene” as an example, whose IUPAC name is “poly(1-phenylethene-1,2-diyl)”, SMILES notation can be “*CC(*)c1ccccc1” and InChI string is “1S/C8H8/c1-2-8-6-4-3-5-7-8/h2-7H,1H2”. Researchers and publications most typically use simply “Polystyrene” or “poly(styrene)” or “PS” or a handful of similar common shorthands to refer to this common chemical compound. A hub that can link the chemical identifiers with the common conventional names that researchers use is in urgent need. Here we present a multi-algorithm-based mapping methodology that is optimized to solve the indexing issue and promote easy data exchange and application of rapidly advancing machine learning methods to polymer systems.

To generate insights from data resources, in addition to consistent identifiers, computational methods and algorithms require large scale, efficient data exchange. Application Programming Interface (API) is a concise solution. A lightweight REpresentational State Transfer (RESTful) API simplifies the user interaction with the hub to an HTTP request and a return. APIs are widely used in many data resources in the materials design domain [17–19]. In this work, we propose ChemProps, a RESTful API enabled database that takes in common polymer names and returns chemical identifiers (unique SMILES) with tolerance of expression differences as mentioned earlier. The rest of the paper is as follows. We describe the structure of ChemProps API, data storage/update protocols of ChemProps database, and how we optimize the polymer name mapping algorithms in detail in “Methods” section. The results of optimization and examples of how to use ChemProps either through HTTP requests or GUI are given in “Results” section. We discuss the potential uses of ChemProps in “Discussion” section and summarize in “Conclusion” section.

## Methods

### Structure of ChemProps API

**Fig. 1:**
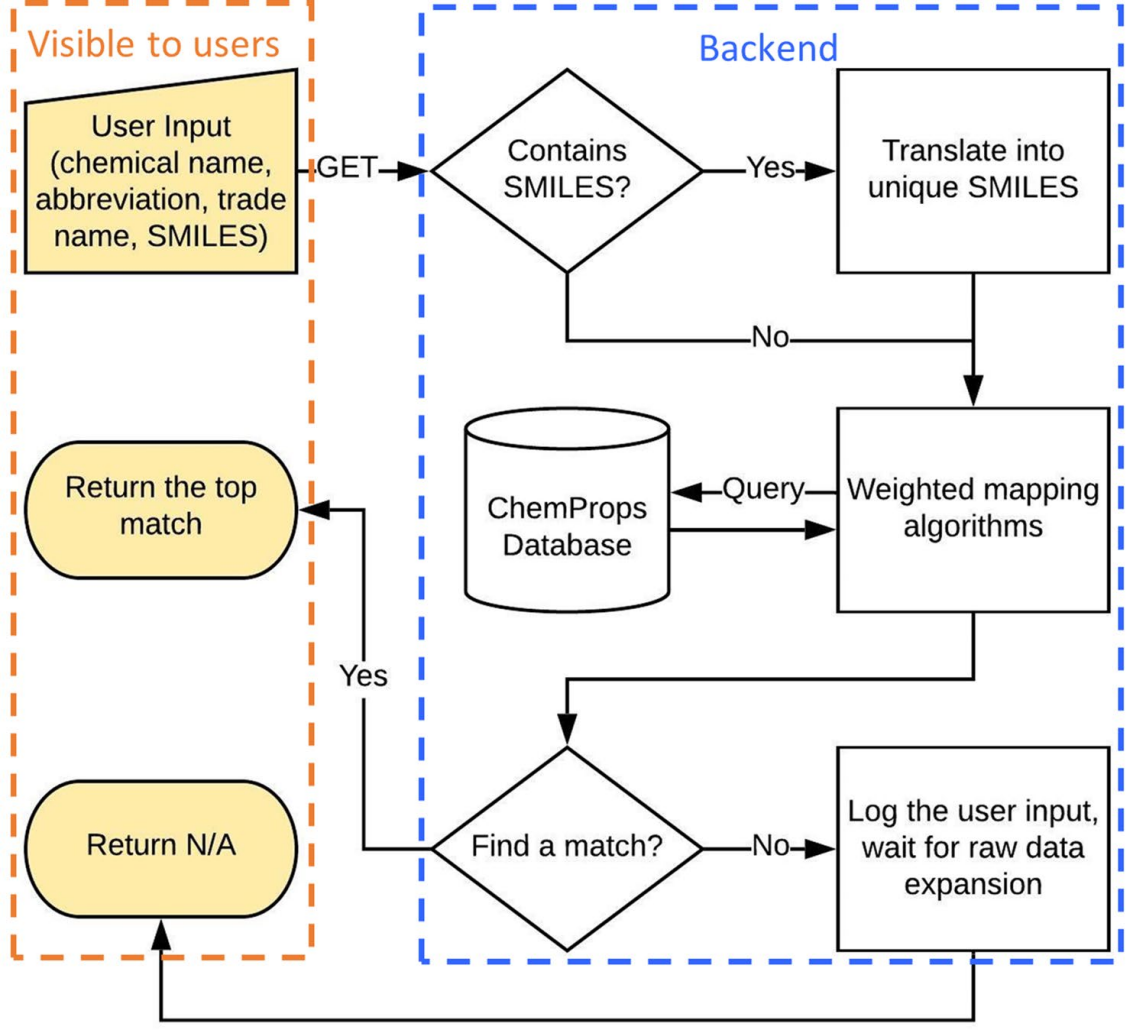
The structure of the ChemProps API

Table 1 shows the input names and descriptions of a GET request. To use the services, users only need to refer to Table 1 to send a GET request to the endpoint at the ChemProps API [20]. We would like to highlight that only the *polfil* and the *ChemicalName* parameters are required inputs. Users can combine these two parameters with any combinations or none of the other three optional inputs to form a valid payload to the ChemProps API. The results will be returned within seconds with the format given in Table 2. Figure 1 shows the overall structure of the ChemProps API for polymer name standardization. Please note ChemProps is designed for standardizing both polymer names and composite nano-filler names. The *polfil* parameter, which states whether users search for a polymer name or a filler name, is assumed to be “pol” throughout this paper.^2^ In Fig. 1, only the yellow part is visible to users, showing the essence of a lightweight RESTful API, while the backend scripts carry all the loads. In the backend process, first a script will check for the existence of *SMILES* parameter in the input and send it to the SMILES translation module for standardization, if present. The SMILES translation module sends the input SMILES string to the NIH Online SMILES Translator to generate the Kekule unique SMILES (uSMILES) representation [21]. This module can remove flavors from SMILES and unify them in most cases. The details of the uSMILES generation can be found in the work by Weininger et al. [22] After this step, the input package moves to the algorithms section, which is the heart of ChemProps. A set of internal evaluation queries are designed to represent a predefined set of mapping algorithms. Each query, which implements a single algorithm, may find multiple results or may not find any results within the ChemProps core database. To ensure the reliability of our service, we assign an optimized weight, which will be updated over time as new polymers are included into the system, to each query and let them vote for the result by summing up the weights of each returned chemical. We describe the optimization process in a later section in detail. The chemical with the highest sum of weights will be returned. There are two cases when we will log the input and notify the admin. First is when no results can be found by any queries, which means ChemProps might need to welcome new chemicals. Second is when two chemicals tie at the top, which we try to avoid through optimization.

**Table 1:** A list of input information of a GET request

Name	Type	Description	Example
*polfil*	String	Search type: Use “pol” for polymer or “fil” for filler	*“polfil”: “*pol”
*ChemicalName*	String	Chemical name to locate	*“ChemicalName”: “*polystyrene”
*Abbreviation*	String	Optional abbreviation to locate	*“Abbreviation”: “*PS”
*TradeName*	String	Optional trade name to locate	*“TradeName”: “*Styrofoam”
*SMILES*	String	Optional specific SMILES value to locate	“SMILES”: “*CC(*)c1ccccc1”

**Table 2:** A list of returned information of a GET request if successful (Code 200)

Name	Type	Description	Example
*StandardName*	String	Standard chemical name	*“StandardName”*: “Polystyrene”
*density*	String	Chemical density at 25 °C in g/cm^3^	*“density”: “*1.04”
*uSMILES*	String	Polymer unique SMILES	*“uSMILES”*: “C(C(C1 = CC = CC = C1)[*])[*]”

### Data

The core ChemProps database was built on MongoDB, a NoSQL database. As a starting point, we begin with a core subset of all possible polymers to create a set of robust algorithms that are continuously calibrated to optimal performance with the introduction of more polymers into ChemProps. Raw data is stored on the cloud in a Google spreadsheet for stability. We pulled 515 polymer names, considered as standard names, from Polymer Genome [23] along with associated SMILES and density information as the skeleton of the raw data. We adopted the customization of marking the linking atoms of the polymer chain with [*] by Polymer Genome. We then augmented the raw data with 49 polymer names pulled from NanoMine for a total of 564 unique polymer names sitting in the Google spreadsheet. Of these 564 unique polymer names, 89 polymer names are fully indexed with their unique SMILES, abbreviations, synonyms, trade names and density information, which are used in the rest of this current study. All SMILES notations were converted to unique SMILES by the translation module [24]. Additional information was collected from sources including MatWeb [25], Polymer Database [26], and Polymer Science Dictionary [27] semi-automatically. Overtime, more of the 564 polymer names will become fully indexed. 54 fillers (such as silica or carbon nanotubes) are also indexed in ChemProps with their synonyms and density information.

**Fig. 2:**
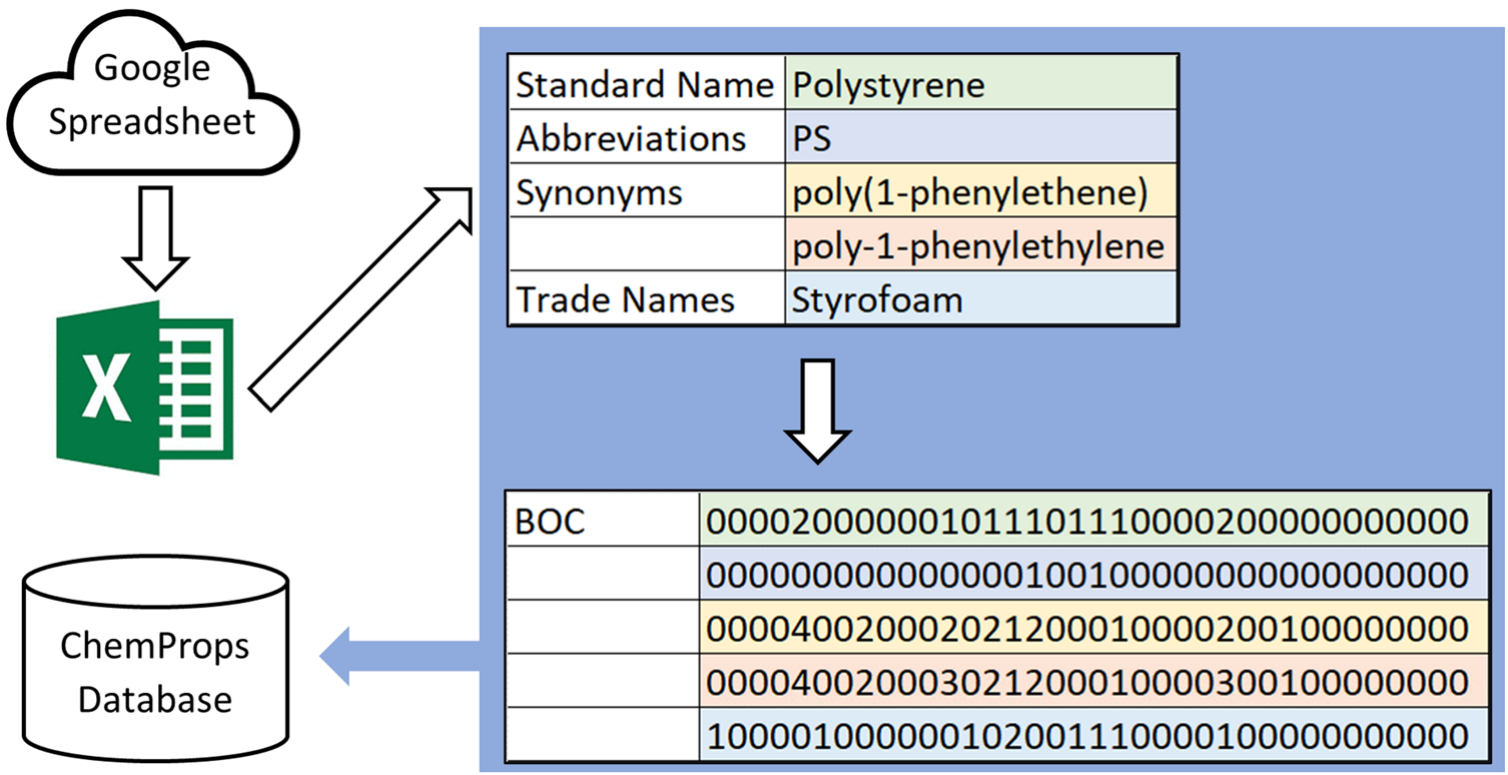
Illustration of the data ingestion process, BOC correspondence are color coded

To begin the algorithm development, we borrow the idea of bag-of-words (BOW) [28] from natural language processing to build a bag-of-characters notation (BOC). BOC is a string of digits with its first 26 digits indicating the occurrence of 26 alphabetic characters from a to z and its last 10 digits representing the occurrence of 10 Arabic numerals from 0 to 9. We cap the occurrence of a character at 9 in its BOC string since it is not common for a character or numeral to occur more than 9 times in a polymer name. All strings are converted to lowercase before transforming into BOC strings. The merits of BOC include disregarding the character order and ignoring non-alphabetic and non-digit characters like whitespaces, dashes, and parentheses. These merits are especially useful for polymer name translation as minor changes in spacing and hyphenation are common in polymer representation. We also introduce a BOC-alph representation which is an alphabet only version of the BOC string. Upon ingesting data from the cloud into the database, a script will convert standard names, abbreviations, synonyms, and trade names into BOC strings for storage. Figure 2 shows an example of the ingestion and conversion process. On the cloud side, since the skeleton already covers chemical names, SMILES and density, an administrator only needs to fill in the common abbreviations, synonyms, and trade names to “activate” a new polymer. A python script is called automatically every week to back up the spreadsheet from the cloud, scan inside the 564 polymers for all the “activated” polymers that have either abbreviations, synonyms or trade names filled in, which currently numbers 89, format the data, and push into the MongoDB for update. This design makes it easy to update the ChemProps database both in depth and in width, which is critical for a growing database, by only interacting with the Google spreadsheet. In depth, administrators can add more polymers by simply add a row in the Google spreadsheet. In width, a new property such as glass transition temperature can be added to the ChemProps database by adding a column in the Google spreadsheet and a few more lines in the digesting scripts.

### Weighted voting algorithm for name mapping

**Fig. 3:**
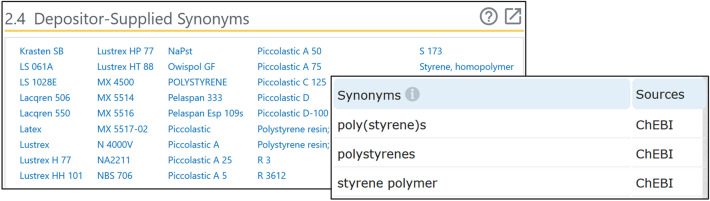
Screenshots of synonyms of polystyrene on PubChem [33] and ChEBI [34]

The conventional way of dealing with synonyms is the brute force approach, which simply tabulates all known synonyms as shown in Fig. 3. This method, however, is unstable and still subject to errors as new differences in expression are encountered. Based on our experience with the 1500 + samples in NanoMine, we propose 12 mapping algorithms to vote for the final match given the user input. Table 3 summarizes the proposed algorithms.

**Table 3:** Summary of the proposed mapping algorithms

Algorithm ID	Description
1	Search within unique SMILES with translated *SMILES*
2	Search within standard names with *ChemicalName*
3	Search within abbreviations with *ChemicalName*
4	Search within synonyms with *ChemicalName*
5	Search within abbreviations with *Abbreviation*
6	Search within BOC with BOCed *Abbreviation*
7	Search within tradenames with *TradeName* using relaxed BOW comparison
8	Search within BOC with BOC-alphed *TradeName* by comparing the first 26 digits
9	Search within BOC with BOCed *ChemicalName*
10	Search within BOC with BOC-alphed *ChemicalName* by comparing the first 26 digits
11	Search within standard names with *ChemicalName* using relaxed BOW comparison
12	Search within synonyms with *ChemicalName* using relaxed BOW comparison

We create a variety of BOC and BOW algorithms for high precision polymer name identification that is robust to expression differences. For example, in Table 3 algorithm #6 considers abbreviations like PA 6–6, PA 66 and PA 6/6 as the same. However, the BOC method cannot identify that “Diglycidyl ether of bisphenol-A epoxy resin” and “diglycidyl ether of bisphenol-A” are equivalent. To address this issue, we introduce the relaxed BOW comparison, which is a variation from BOW. We first split the input by non-alphabetic characters to form a bag of words. Then we search the database for entries that contain all the words in the bag. Though the two DGEBA epoxy expressions have different BOC strings, they can be identified by algorithm #12 since the BOW of the second expression is a subset of the first expression. Due to the difference in reliability, we apply a weight to each algorithm and optimize the weights for the best mapping performance. The collection of the weights is denoted as a vector ***w***.

In order to optimize the weights of algorithms, we use the data from NanoMine divided chronologically into training and validation sets. We extract the reported polymer data following the format given in Table 1. We hereby define a searching package (***SP***) as a combination of *ChemicalName, Abbreviation*, *TradeName*, and *SMILES*. The training set contains 166 unique sets of ***SP***s, while the test set contains 54 unique sets of ***SP***s. To start the training process, the initial weight of all 12 algorithms is assigned to one. When a ***SP*** is passed to ChemProps, all 12 algorithms will be used for evaluation one by one. For each algorithm, ChemProps will look at all of the 89 indexed polymers to see if there is a match evaluated through the algorithm using the terms in the ***SP***. Intuitively, we create a vector ***R*** of length 12 for each indexed polymer for initialization. We then fill in the nth spot with either 0 or 1 depending on the evaluation using the nth algorithm with 0 indicating not a match and 1 otherwise. When the ***SP*** is evaluated by all 12 algorithms, we will have 89 ***R*** vectors in theory. However, many of the 89 ***R*** vectors are filled with 12 zeros, which are trivial and discarded. For that specific ***SP***, ChemProps only records the indexed polymers with non-trivial ***R*** vectors and computes their scores by taking the dot product of ***w*** and ***R***. Only the non-trivial ***R*** vectors are generated for better computational efficiency and we denote the names of indexed polymers associated with those non-trivial ***R*** vectors as candidate chemical names, one of which must be the ground truth chemical name based on our training data.

In the first pass through ChemProps of the 166 training ***SP***s, 102 ***SP***s get paired with more than one ***R*** vector and 64 ***SP***s get paired with only one ***R*** vector, which we will discuss later. For each of the 102 ***SP***s, we select from the recorded non-trivial ***R*** vectors the ones with score ranked top two. Since there could be a tie in score for ***R*** vectors, we end up collecting 262 ***R*** vectors from the 102 ***SP***s. For this subset of data, our goal is to maximize the score difference between the ground truth chemical name and other top ranked candidate chemical names via an optimal weight vector ***w***. To accomplish this goal, for each of the 102 ***SP***s, we define a vector ***x*** as the difference between the ***R*** vectors of wrongly mapped candidate chemical names and the ***R*** vector of the ground truth chemical name. ***x*** is a vector of length 12 with values of − 1, 0 or 1: − 1 results if the algorithm finds an incorrectly mapped chemical name, 0 results if the algorithm either finds both or finds neither of the correct and the wrongly mapped chemicals, and 1 results if the algorithm only finds the ground truth chemical name. Then the score difference can be represented by the dot product of ***x*** and ***w***. It is worth mentioning that the ***R*** vector of the ground truth chemical name is not necessarily 1 in all spots. For the rest 64 of the 166 ***SP***s, each of them is paired with only one ***R*** vector. Since the associated candidate chemical names are indeed the ground truth chemical name, we denote these 64 ***R*** vectors as ***Rc*** with letter “c” standing for “correct”.

**Fig. 4:**
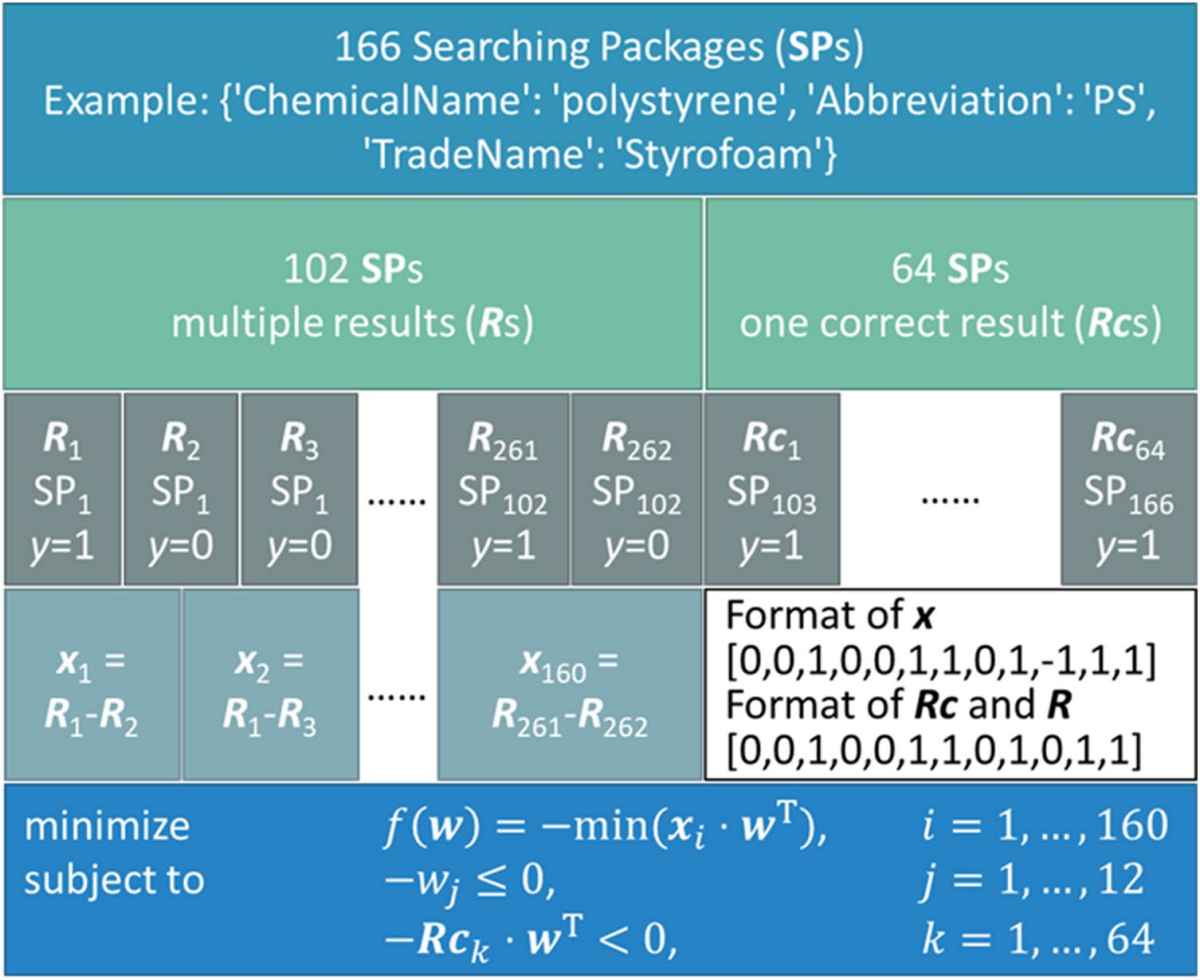
Structure of training data for weights optimization

An illustration of the training data structure is shown in Fig. 4. From top down it shows the breakdown of the training data set. The grey blocks give example for ***R*** vectors and ***Rc*** vectors. *y* being 1 stands for the ground truth chemical name and being 0 stands for the wrong mapping. From ***SP***_1_ to ***SP***_102_, ChemProps returns multiple candidate chemical names meaning multiple ***R*** vectors mapped to one ***SP***. Consider ***SP***_1_ as an example, there are three candidate chemical names returned with ***R***_1_ being the ***R*** vector of the ground truth. Our next step is to obtain the difference between the ground truth ***R***_1_ and the wrongly mapped ***R***_2_ and ***R***_3_, leading to the formation of ***x***_1_ and ***x***_2_. ***SP***_103_ to ***SP***_166_ all return the only ground truth forming the 64 ***Rc*** vectors.

We seek to maximize the score difference between the ground truth chemical name and the wrongly mapped candidate chemical names, while maintaining the weights to be non-negative and the score of 64 ***Rc*** vectors to be positive, ensuring that the ground truth chemical name will still be returned when ***w*** is converged. Therefore, we propose the null negative form of the optimization problem as follows:

$${\text{minimize}}\;f(\varvec{w}) = - min(\varvec{x}_{i} \cdot \varvec{w}^{{\text{T}}} ),\quad \;i = 1, \ldots ,160$$

$${\text{subject to}}\; - w_{j} \le 0\quad j = 1, \ldots ,12$$

$$- {\varvec{Rc}}_{k} \cdot {\varvec{w}}^{{\text{T}}} < 0,\quad k = 1, \ldots ,64$$

**Fig. 5:**
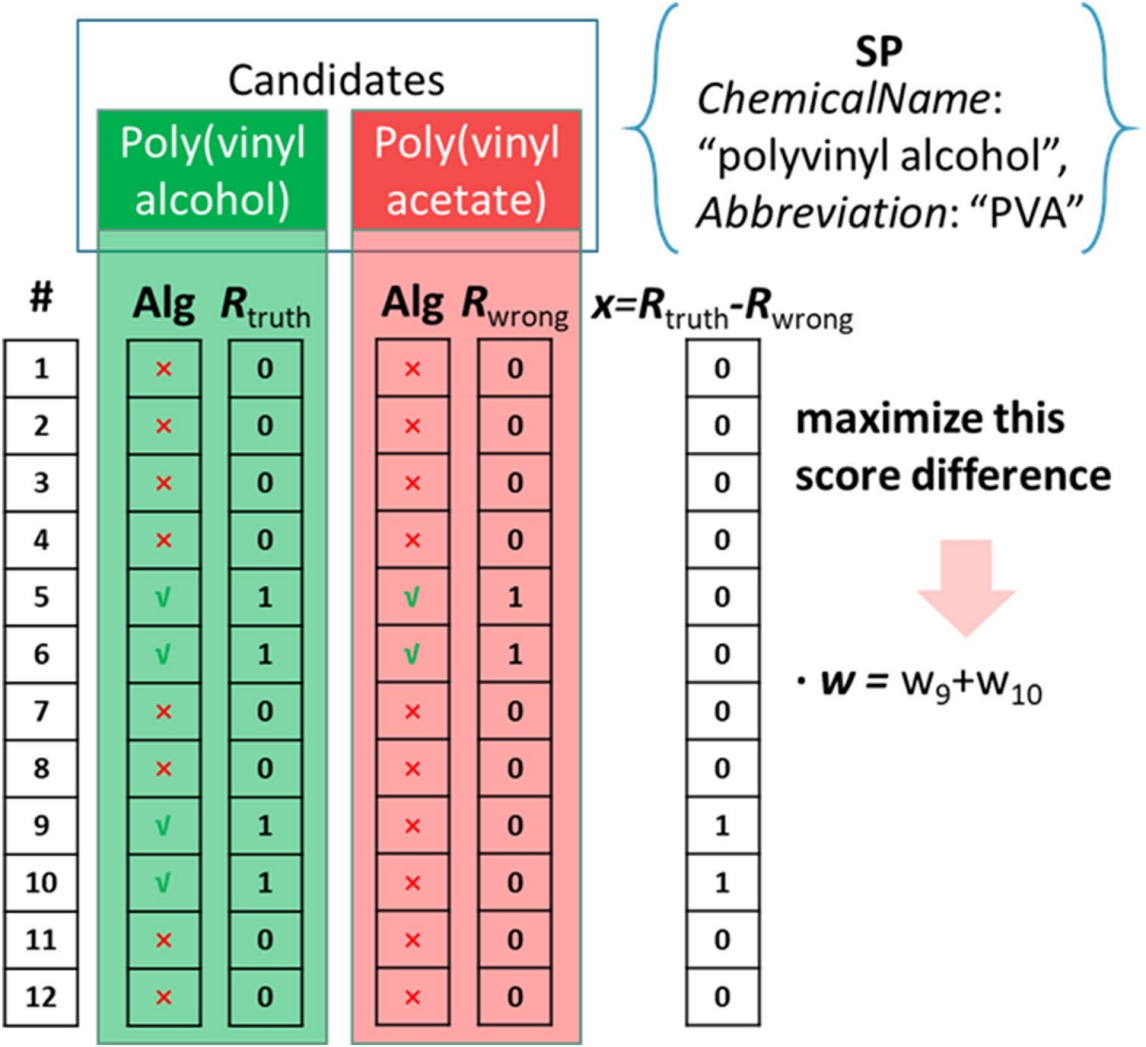
Example of the score difference derivation

The dot product of ***x*** and the weight vector ***w*** is the score difference that we would like to maximize, where a positive score difference indicates that the ground truth chemical name has a higher score than the other candidates and thus will be returned by ChemProps. Instead of maximizing the score difference in the entire training set, we can reduce the objective function to only maximizing the minimum value of all the score differences, as stated in the function above. A detailed example of how we obtain the score difference is given in Fig. 5.

The example ***SP*** in Fig. 5 provides “polyvinyl alcohol” as *ChemicalName* and “PVA” as *Abbreviation*. There are two candidates returned, the ground truth Poly(vinyl alcohol) and the wrongly mapped Poly(vinyl acetate). Since both candidates can be abbreviated as “PVA”, algorithm #5 and #6 return both candidates. However, algorithm #9 and #10 can only return the ground truth because Poly(vinyl alcohol) has the same BOCed and BOC-alphed chemical name as the input *ChemicalName*. Therefore, the score difference is the sum of the weights assigned to algorithm #9 and #10, i.e. ***w***_9_ and ***w***_10_. It is worth noting that algorithm #2 does not find the ground truth due to the existence of the parentheses in the standard name highlighting the need for algorithms #9 and #10 using BOC.

We select the widely used Generalized Reduced Gradient (GRG) nonlinear method for optimization. We also apply a ten-fold validation to the optimization problem in which the 160 ***x***’s are randomly divided into ten groups. For each iteration, we omit a group of 16 ***x*** vectors and conduct optimization on the ***w*** vector using the remaining 144 ***x*** vectors. We then use the omitted ***x*** vectors for validation each time and enforce that the minimum score of the omitted ***x*** vectors must be positive to pass. This process is repeated ten times.

## Results

### Optimization

The results of the optimization and ten-fold validation are summarized in Table 4.

**Table 4:** Results of weight vector optimization and ten-fold validation

Fold	***w***_1_	***w***_2_	***w***_3_	***w***_4_	***w***_5_	***w***_6_	***w***_7_	***w***_8_	***w***_9_	***w***_10_	***w***_11_	***w***_12_	Min training score	Ten-fold test score
1	1	1	1	1	0.8	0.8	0	0	1.2	1.2	1	2	0.8	0.8
2	1	1	1	1	0.8	0.8	0	0	1.2	1.2	1	2	0.8	2.4
3	1	1	1	1	0.8	0.8	0	0	1.2	1.2	1	2	0.8	2.4
4	1	1	1	1	0.8	0.8	0	0	1.2	1.2	1	2	0.8	0.8
5	1	1	1	1	1	1	0	0	1	1	1	2	1	0
6	1	1	1	1	0.8	0.8	1	1	1.2	1.2	1	1	0.8	− 1
7	1	1	1	1	0.8	0.8	0	0	1.2	1.2	1	2	0.8	0.8
8	1	1	1	1	0.8	0.8	0	0	1.2	1.2	1	2	0.8	2
9	1	1	1	1	0.8	0.8	0	0	1.2	1.2	1	2	0.8	1.6
10	1	1	1	1	0.8	0.8	0	0	1.2	1.2	1	2	0.8	1.6

The ***w*** values in Table 4 are the converged weight factors. For each iteration, the minimum training score is the minimum score difference in the 144 ***x*** vectors used for optimization, while the ten-fold test score is the minimum score difference in the omitted 16 ***x*** vectors used for validation. The results show that all but iterations 5 and 6 converge to a consistent set of weight factors w* = {1, 1, 1, 1, 0.8, 0.8, 0, 0, 1.2, 1.2, 1, 2} with positive ten-fold test score. In contrast, the ten-fold test score of iteration 5 and 6 are non-positive, which means that within the 16 omitted cases, at least one wrongly mapped chemical name has a score that is higher or equal to the ground truth. If we adopt the converged weight factors in iterations 5 and 6, we know that ChemProps will return wrong chemical names in these cases. Our next step is to check the validity of w* over the training data in iterations 5 and 6 by recomputing the minimum training score and the ten-fold test score of the two iterations when the original converged weight factors are replaced with w*. Positive values for both updated scores indicate that we can consider w* a feasible solution to our optimization problem on the training set. w* works for the iteration 5, whose min training score decreases from 1 to 0.8 and ten-fold test score increases from 0 to 0.8. w* also works for the iteration 6, whose min training score remains the same at 0.8 and ten-fold score increases from − 1 to 1.6. Therefore, we have shown that w* is a feasible solution on the training set.

The final step is to verify that w* also works for the 54 ***SP***s from the test set. We configure ChemProps algorithm section to use w* and feed the 54 ***SP***s to the API. All ***SP***s have the correct mapping returned, indicating w* is a feasible solution to our optimization problem. Note that w* assigns zero weight to algorithm #7 and #8, which are *TradeName* related. It shows trade names are at this point not an important mapping feature. However, we retain them in the overall framework as the weight factors will continue to evolve through the presented optimization pipeline as ChemProps grows. It is also noted that due to the source of NanoMine data being mostly published literature, none of the current ***SP***s, training or test, contain *SMILES*. That is why the weight of algorithm 1 is the same as the initial value in any iteration. We assume *SMILES* as a chemical identifier is reliable if provided. We keep the weight for algorithm #1 as one and as more data enters the system with SMILES fields, the coefficients will evolve.

### Example of API access for ChemProps

In this example we introduce how to use python to access ChemProps API. More examples for accessing by Javascript, Java, Go and PHP are provided (see Additional file 1). The package requirement includes requests, which can be installed through “pip install requests” in the command line. Users must login into NanoMine platform by InCommon or OneLink accounts to request for tokens needed for API calls. Procedures to apply for the account and to request for tokens are provided elsewhere (see Additional file 1). Once the token is obtained, users can use the following scripts to use ChemProps API and search, for example, polystyrene:
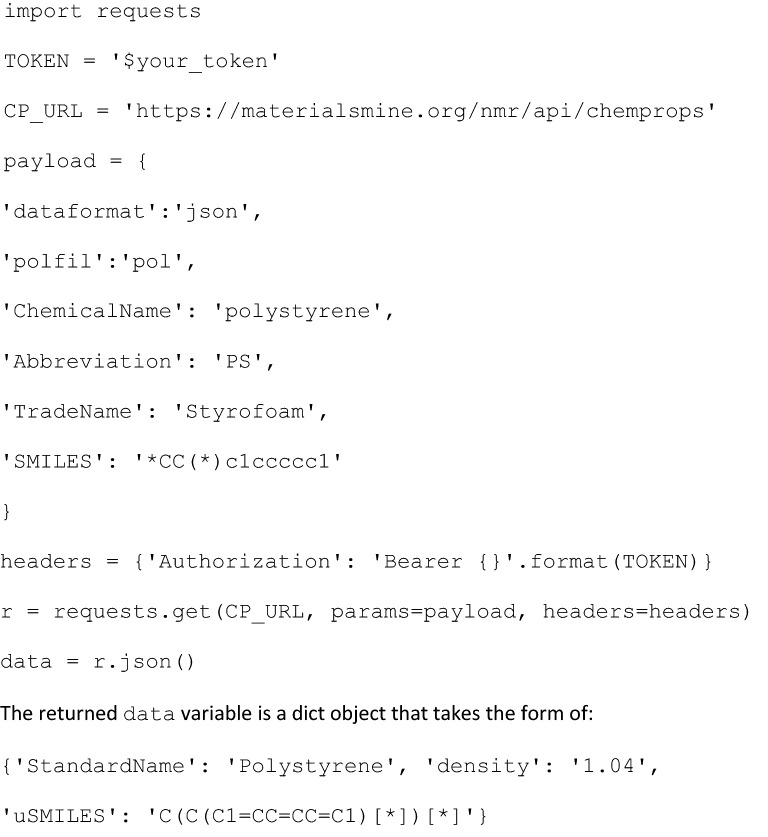


As guided in Table 1, users can replace the corresponding payload parameters with their values. Optional parameters like *Abbreviation*, *TradeName*, and *SMILES* can be removed from the payload in the previous example.

### Example of GUI access for ChemProps

**Fig. 6:**
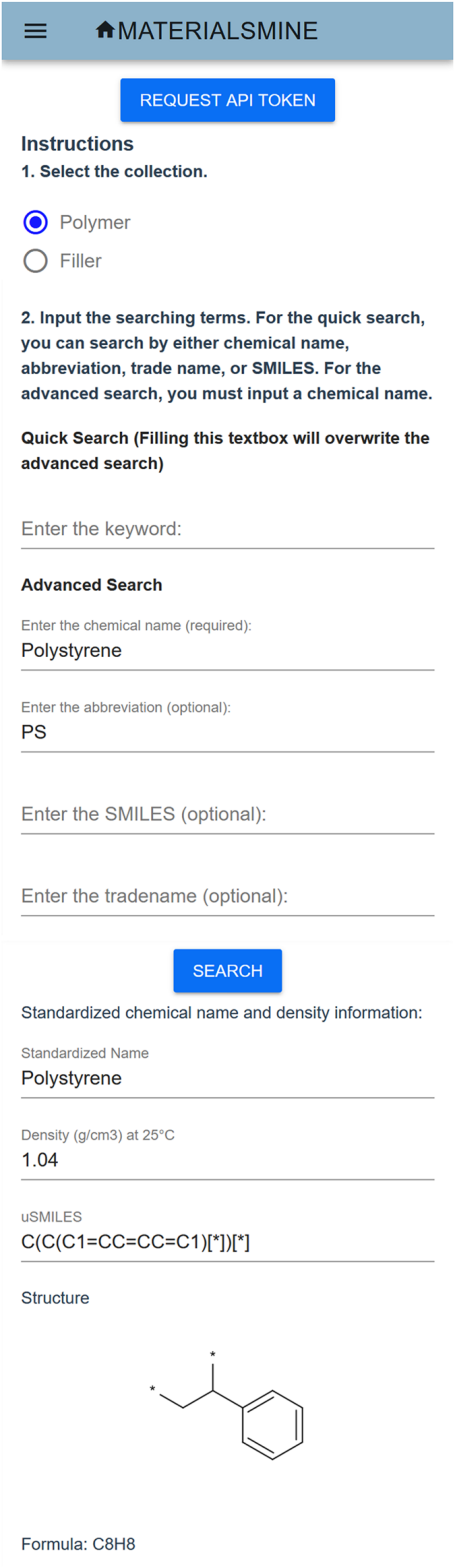
The ChemProps Graphical User Interface [35]

In addition to the ChemProps API, we also developed a Graphical User Interface (GUI) for users that are not familiar with API calls. We make the GUI open for users without an InCommon or OneLink account. Figure 6 shows the ChemProps GUI.

Users can select search for either polymer or filler and put the keywords into the corresponding text boxes. For polymer search, a convenient quick search box is enabled, where users can input any one of the chemical name, abbreviation, SMILES or trade name to do the search. An extra feature as compared to the API call is that the chemical structure and formula [29] will be displayed on the page when a result is found in ChemProps polymer database. If no results are found in ChemProps, the page will display a warning message “No results found. Admin alerted to update the database. Please try again in a week.” with an error code 404.

## Discussion

ChemProps is a hub that acknowledges the large number of variations of names for polymers, merges those aliases in an intelligent and systematic way, and speaks the languages that both computer and materials scientists understand. Apart from its potential in Materials Informatics in the future, it can be useful to the materials science community immediately. Similar to the example of Reaxys, widely used chemical databases like ChEBI use text search for chemicals by default, which brings inconsistency in the results.^3^

With ChemProps, those chemical databases can comb their polymer data and remove the duplicates as needed.

Another immediate usage of ChemProps is to convert common polymer names to uSMILES. Since the introduction of SMILES, major polymer related chemical databases gradually support the more accurate “search by SMILES” function. However, many researchers do not read or speak SMILES. This language barrier stops users from utilizing the more advanced searching functionalities. ChemProps can act as a bridge to enable general chemical name querying in those databases instead of using the complicated SMILES. A general workflow could be (1) users input chemical names in the search box of those databases as usual (2) the search function makes an API call to ChemProps (3) ChemProps returns the result including the uSMILES (4) the search function uses the returned uSMILES for accurate searching. We must point out that since SMILES are software generated, it has different flavors. To reduce the impact of those software generated flavors, ChemProps uses uSMILES throughout the system. So ChemProps returns uSMILES only. To use ChemProps as a bridge for querying by SMILES, it is recommended that our potential collaborators also use uSMILES generated by the SMILES translation module of ChemProps to be consistent with ChemProps such that the returned value of an API call to ChemProps can be used in query directly.

Beyond the original purpose of ChemProps, we have discovered that a robust solution to the polymer indexing problem in NanoMine has led to the solutions of related problems. For example, the amount of filler in a composite system can be expressed by mass fraction or volume fraction, which are convertible if the densities of the polymer matrix and the filler are available. However, such data are not typically provided in publications. By standardizing the polymer and filler names, we have been able to use ChemProps to automatically populate the density data to initiate the mass fraction – volume fraction conversion process. Now that the naming issue is solved, there are yet other automation agents can be enabled beyond the mass fraction – volume fraction conversion agent. For example, an autonomic inference agent that populates storage modulus and loss modulus for polymers can be written readily to deploy. Single-value properties that do not vary significantly within the same polymer class like dielectric constants in the polymer database prepared by Huan et al. [30] can also be readily integrated into ChemProps thanks to its ability to resolve polymer names in different forms and flexible data ingestion design. Beyond properties, we can couple with another effort to produce better polymer indexing entitled BigSMILES [16] by ingesting the BigSMILES representations that were specifically designed for polymer systems into the ChemProps database to provide another machine-readable representation in the output of the ChemProps API.

An emerging machine-readable format of chemical mixtures are valuable to Materials Informatics, especially polymer composite data resources like NanoMine since mixtures have a significant presence throughout the processing steps [31]. However, currently polymers need to be defined by names or database identifiers due to the size of macromolecules as stated by the authors. ChemProps could help with the issue since it standardizes polymer names and provides the associated uSMILES, which is machine-readable. In line with the effort to describe the processing steps, a recent work named PolyName2Structure [32] seeks to convert polymer name representations to monomer structures, predict the polymerization pathway and identify the reaction groups. In addition to IUPAC names and source-based names, PolyName2Structure also accepts common names by passing it to an in-house dictionary to resolve the structures. PolyName2Structure can expand the range of feasible common names by making an API call to ChemProps.

## Conclusion

In this work, we propose a twelve algorithm based mapping methodology named ChemProps that is optimized to solve a polymer indexing issue which routinely impedes the progress of Materials Informatics for polymeric based systems. ChemProps acknowledges the large number of variations of names for polymers, merges those aliases in an intelligent and systematic way, and speaks the languages that both computer and materials scientists understand. A cloud-based design makes ChemProps not only reliable but also easy for admins to add data and expand properties. With the RESTful API, users can access the powerful service with a few simple lines of HTTP request or through the user-friendly graphical user interface powered by ChemProps API with quick search function enabled.

To ensure the accuracy of ChemProps, we assign a weight factor to each algorithm to generate scores for candidate chemical names and optimize them using the data from NanoMine. We favor the cases when ground truth chemical names have a higher score than other candidate chemical names as the correct mapping will be returned. Therefore, our goal is to maximize the score difference between the ground truth and other candidate chemical names. To further reduce the computational problem, we maximize the minimum value of the score difference between the ground truth chemical name and the other candidate chemical names. Ten-fold validation is utilized on the training data points to prevent overfitting issues. We configured ChemProps with the converged set of weight factors using training data and tested it on the test-set searching packages. A 100% test accuracy is achieved. The weight factors of two algorithms related with trade names currently converge to zero, showing that trade name might not be an important mapping feature at present. However, we retain the two algorithms in the overall framework since the weight factors will evolve through the same optimization pipeline as ChemProps grows. In the current set of data, we use 166 searching packages (each corresponding to a unique polymer) as training data, retaining 54 searching packages for testing. It is important to note that ChemProps is continuously growing and the living algorithm presented here will be continuously run to update the weighting functions as new polymers are added to the system.

We believe ChemProps can contribute to the community in several ways. First, other polymer databases can use ChemProps to comb their data and remove duplicate entries. Second, other polymer databases that enables the more accurate “search by SMILES” function can allow users to input common human-readable names while using ChemProps as a translator through API calls to conduct SMILES search in the backend. Third, the easy-to-update design makes ChemProps a good tool to auto-populate polymer properties, which is useful to enable a growing array of automated agents for materials data resources.

## Supplementary Information

**Additional file 1.** Detailed instructions on how to access ChemProps API.

## Data Availability

The source code of ChemProps and a copy of the raw data are openly available at https://github.com/Duke-MatSci/ChemProps. The ChemProps GUI is available at https://materialsmine.org/nm#/ChemProps.

## Abbreviations

PMMA: Poly(methyl methacrylate)
IUPAC: International Union of Pure and Applied Chemistry
SMILES: Simplified Molecular-Input Line-Entry System
InChI: International Chemical Identifier
API: Application Programming Interface
REST: REpresentational State Transfer
uSMILES: Kekule unique SMILES
BOW: Bag-Of-Word notation
BOC: Bag-Of-Character notation
SP: Searching Package
GRG: Generalized Reduced Gradient
GUI: Graphical User Interface

## Footnotes

1 Results for “polymethyl methacrylate” counts 3974, while results for Poly(methyl-methacrylate) counts 1 on Reaxys. Note that the quotation marks here are part of the searching term.

2 The “fil” option for filler search will be described elsewhere.

3 5 entries were found by searching for Poly(methyl-methacrylate), while 52 entries were found by searching for polymethyl methacrylate on ChEBI.
