# Supplementary material for: ChemProps: A RESTful API enabled database for composite polymer name standardization
Source: J Cheminform. 2021 Mar 12;13:22. doi: 10.1186/s13321-021-00502-6 (PMC7955638; doi:10.1186/s13321-021-00502-6)
Supplement: Supplementary file 1 — Additional file 1. Detailed instructions on how to access ChemProps API. [file 13321_2021_502_MOESM1_ESM.docx]

**Supplementary Material**

**ChemProps: A RESTful API enabled database for composite polymer name standardization**

Bingyin Hu, L. Catherine Brinson

Department of Mechanical Engineering and Materials Science, Duke University, Durham, North Carolina 27708, United States

Corresponding author: L. Catherine Brinson Email: cate.brinson@duke.edu

This document provides text and figures pertaining to (1) procedures to Duke OneLink account applications; (2) procedures to request for tokens needed for ChemProps API; (3) example for accessing ChemProps API by Javascript; (4) example for accessing ChemProps API by Java; (5) example for accessing ChemProps API by GO; (6) example for accessing ChemProps API by PHP.

1. **Duke OneLink account application**

Users can go to <https://accounts.oit.duke.edu/onelink/register> to apply for a Duke OneLink account. Without a Duke OneLink account or InCommon account (under development), users may not be able to apply for the API token and thus cannot use ChemProps service through API calls. Figure S1 shows the landing page of the Duke OneLink account registration.


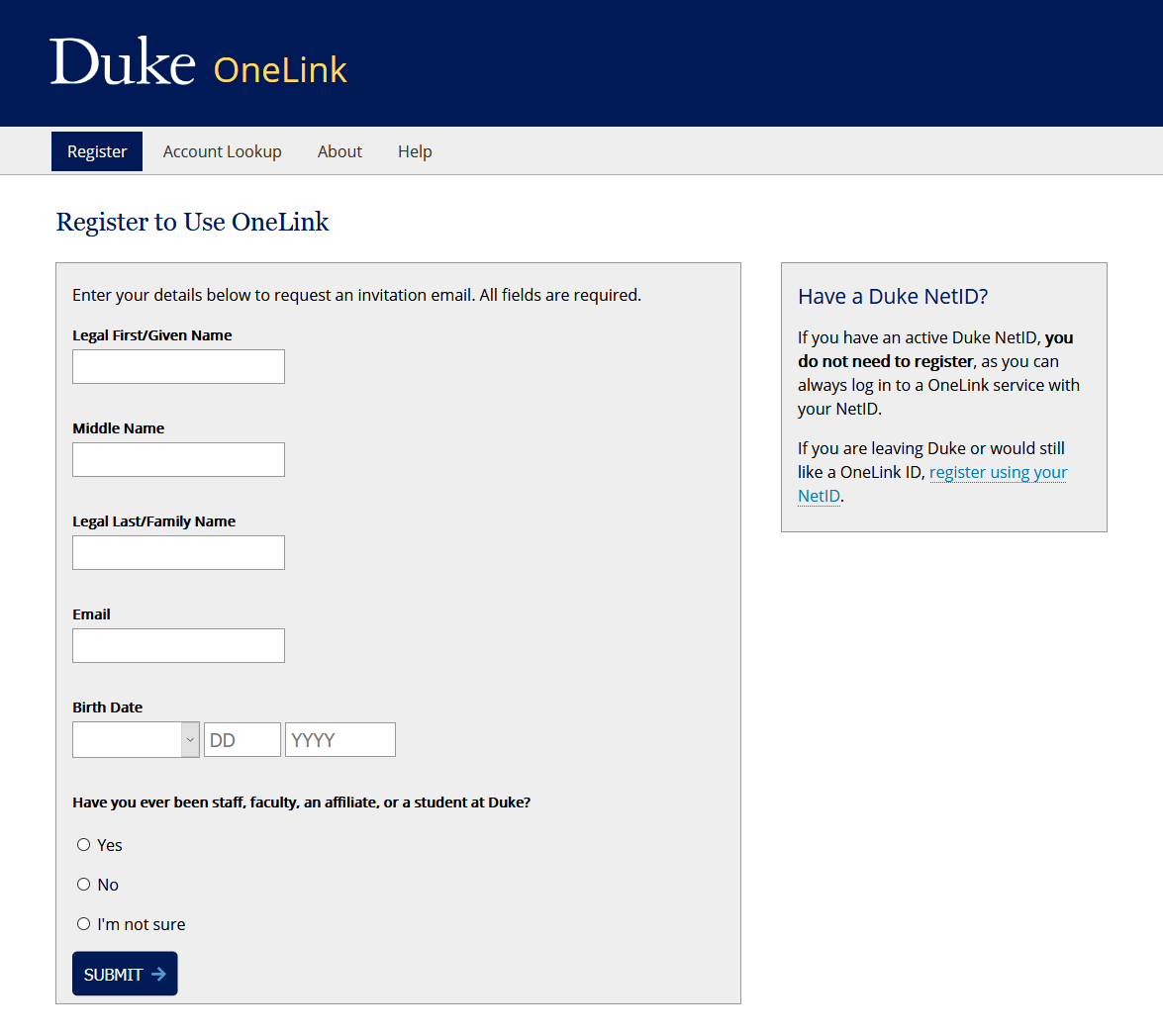


Figure S1. The landing page of Duke OneLink account registration

1. **ChemProps API token request**

To use the ChemProps API, users must go to the ChemProps GUI at <https://materialsmine.org/nm#/ChemProps> first to request a token. This can be done by clicking on the “REQUEST API TOKEN” button located at the top of the ChemProps GUI page as shown in Figure S1 or directly by the URL <https://materialsmine.org/nm#/ChemPropsAPIToken>.


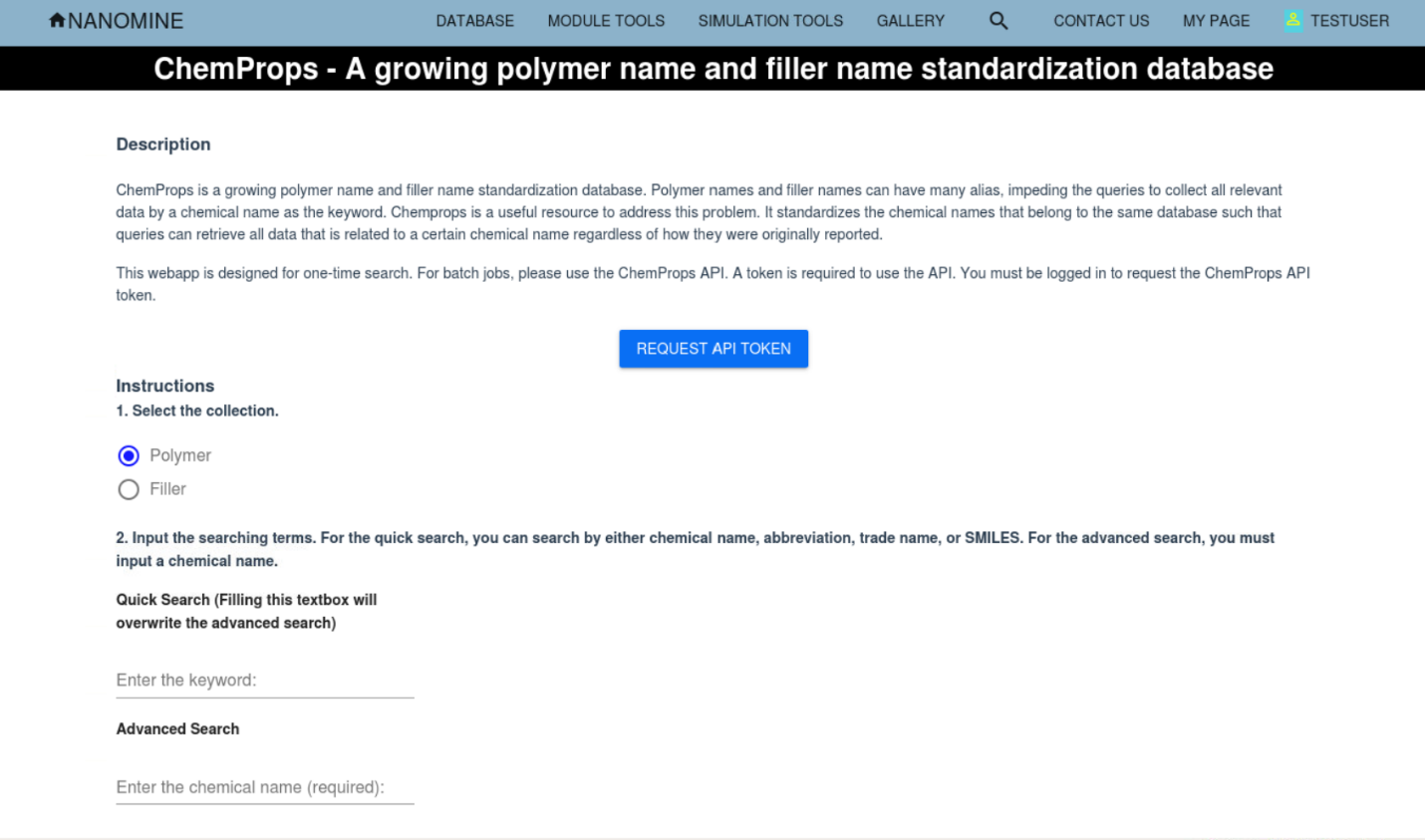


Figure S2. Illustration of the request API token button on the ChemProps GUI page

If users are not logged in on NanoMine through OneLink or InCommon account, an alert message will display for redirection to login or OneLink account registration as shown in Figure S3.


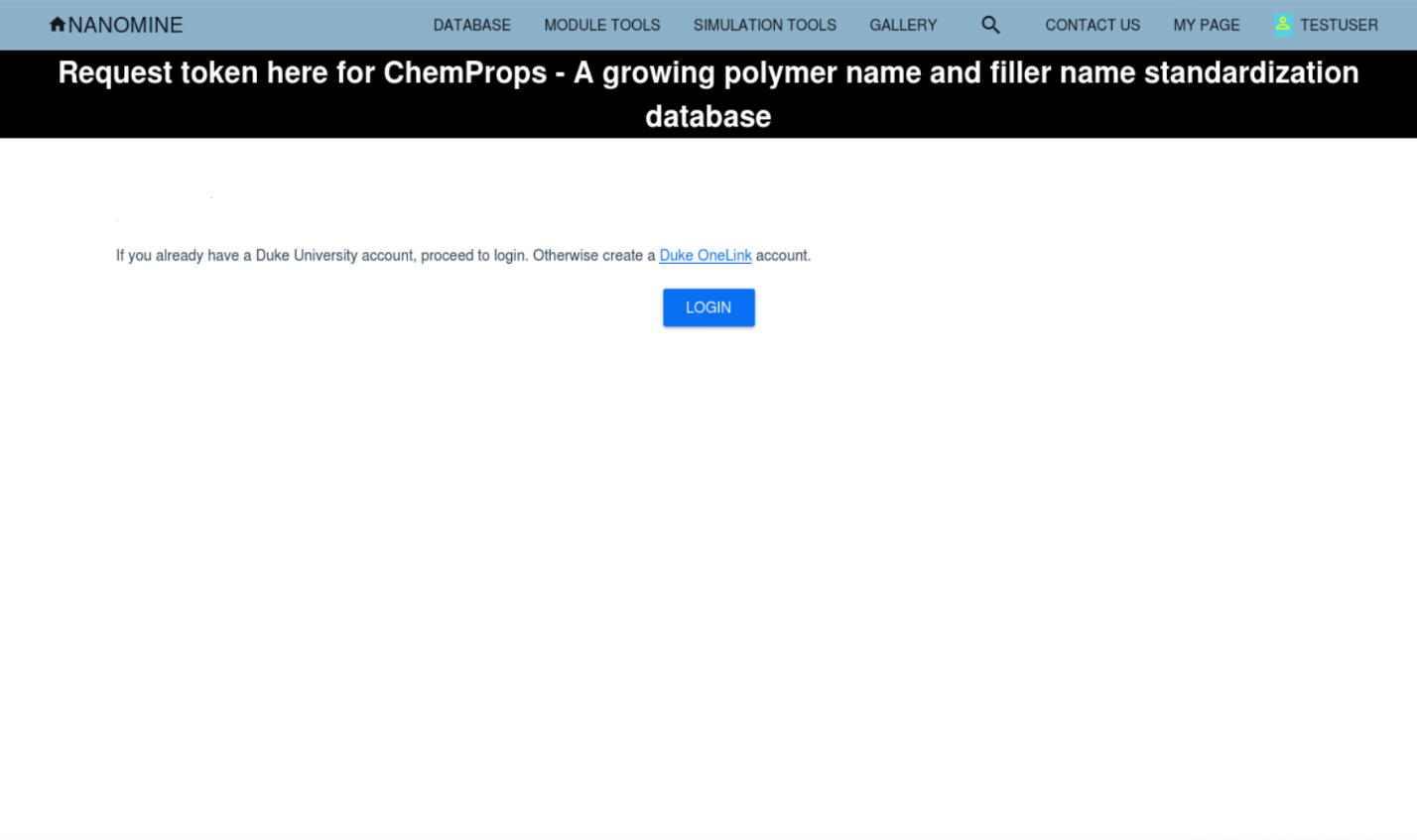


Figure S3. Example token request page if users not logged in

If users have already logged in and they have not requested for a ChemProps API token before, they will be asked to provide a domain secret as shown in Figure S4.


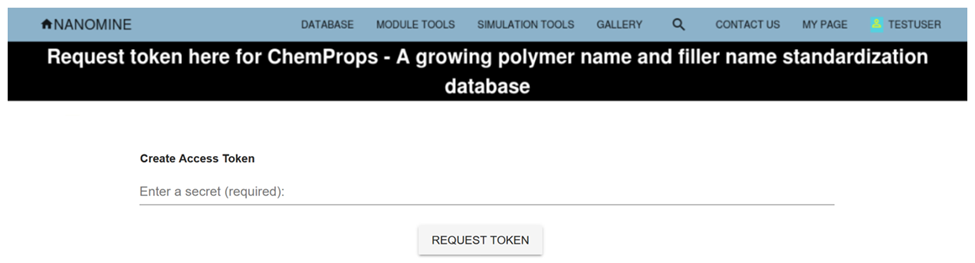


Figure S4. Example token request page for first-time logged-in users

Then the “REQUEST TOKEN” button will be activated. Clicking on the button and the token will display as shown in Figure S5.


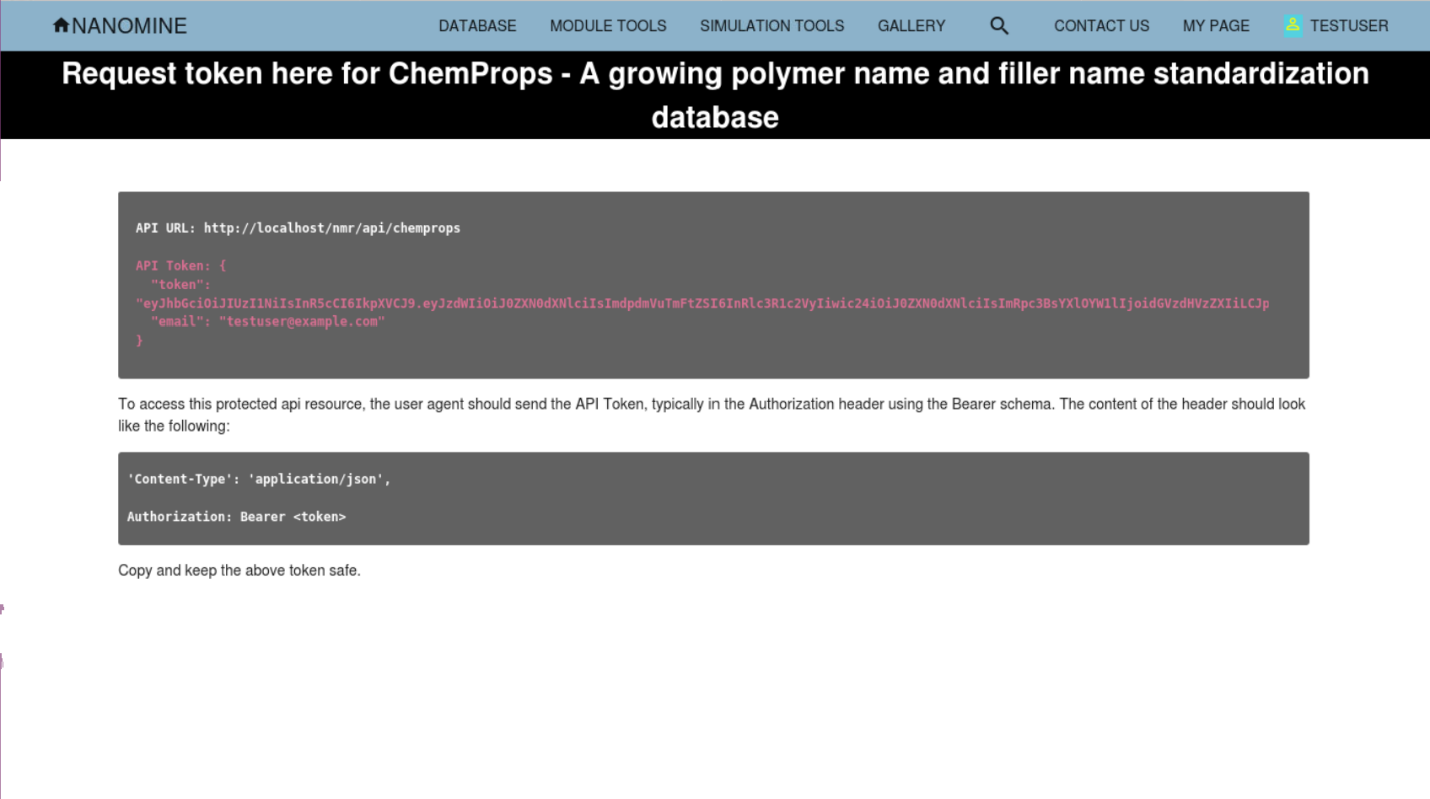


Figure S5. Example for a successfully requested API token

Users should keep their token safe. In case users forgot their token, the same message containing the token information will display if users log back into the system and navigate to the token request page.

1. **Example Javascript code for using ChemProps API**

Assume the token for all examples is Bearer OiJIUzI1NiIsInR5cCI6IkpXVCGxlLmNvbSIsImlzQW5vbnltb3VzIjpmYWxzZSwidXNlckV4aXN0cyI6dHJ1ZSwia. Assume the input parameters are the same as the python example given in the main paper.

var request = require(‘request');

var options = {

'method': 'GET',

'url': 'https://qa.materialsmine.org/nmr/api/chemprops?ChemicalName=polystyrene&Abbreviation=PS&TradeName=Styrofoam&SMILES=*CC(*)c1ccccc1&polfil=pol',

'headers': {

'Authorization': 'Bearer OiJIUzI1NiIsInR5cCI6IkpXVCGxlLmNvbSIsImlzQW5vbnltb3VzIjpmYWxzZSwidXNlckV4aXN0cyI6dHJ1ZSwia'

}

};

request(options, function (error, response) {

if (error) throw new Error(error);

console.log(response.body);

});

1. **Example Java code for using ChemProps API**

OkHttpClient client = new OkHttpClient().newBuilder().build();

Request request = new Request.Builder()

.url("https://qa.materialsmine.org/nmr/api/chemprops?ChemicalName=polystyrene&Abbreviation=PS&TradeName=Styrofoam&SMILES=*CC(*)c1ccccc1&polfil=pol")

.method("GET", null)

.addHeader("Authorization", "Bearer OiJIUzI1NiIsInR5cCI6IkpXVCGxlLmNvbSIsImlzQW5vbnpmYWxzZSwidXNlckV4aXN0cyI6dHJ1ZSwia")

.build();

Response response = client.newCall(request).execute();

1. **Example GO code for using ChemProps API**

package main

import (

"fmt"

"strings"

"net/http"

"io/ioutil"

)

func main() {

url :=

"https://qa.materialsmine.org/nmr/api/chemprops?ChemicalName=polystyrene&Abbreviation=PS&TradeName=Styrofoam&SMILES=*CC(*)c1ccccc1&polfil=pol"

method := "GET"

payload := strings.NewReader("")

client := &http.Client {

}

req, err := http.NewRequest(method, url, payload)

if err != nil {

fmt.Println(err)

}

req.Header.Add("Authorization", "Bearer OiJIUzI1NiIsInR5cCI6IkpXVCGxlLmNvbSIsImlzQW5vbnpmYWxzZSwidXNlckV4aXN0cyI6dHJ1ZSwia")

res, err := client.Do(req)

defer res.Body.Close()

body, err := ioutil.ReadAllres.Body)

fmt.Println(string(body))

}

1. **Example PHP code for using ChemProps API**

<?php

require_once 'HTTP/Request2.php';

$request = new HTTP_Request2();

$request->setUrl('https://qa.materialsmine.org/nmr/api/chemprops?ChemicalName=polystyrene&Abbreviation=PS&TradeName=Styrofoam&SMILES=*CC(*)c1ccccc1&polfil=pol');

$request->setMethod(HTTP_Request2::METHOD_GET);

$request->setConfig(array(

'follow_redirects' => TRUE

));

$request->setHeader(array(

'Authorization' => 'Bearer OiJIUzI1NiIsInR5cCI6IkpXVCGxlLmNvbSIsImlzQW5vbnpmYWxzZSwidXNlckV4aXN0cyI6dHJ1ZSwia'

));

$request->setBody('');

try {

$response = $request->send();

if ($response->getStatus() == 200) {

echo $response->getBody();

}

else {

echo 'Unexpected HTTP status: ' . $response->getStatus() . ' ' .

$response->getReasonPhrase();

}

}

catch(HTTP_Request2_Exception $e) {

echo 'Error: ' . $e->getMessage();

}
